# Supplementary material for: WordCommentsAnalyzer: A windows software tool for qualitative research
Source: F1000Res. 2018 Sep 4;7:536. Originally published 2018 May 3. [Version 2] doi: 10.12688/f1000research.14819.2 (PMC6137411; doi:10.12688/f1000research.14819.2)
Supplement: Supplementary file 3 [file f1000research-7-17614-s0002.tgz › b2a79496-4ada-47c4-9682-a99ce867836d.zip!/Eight Iranian Health Journals/Archives of Rehabilitation_28.docx]

© Archives of Rehabilitation. This work is licensed under CC BY-NC 4.0 (https://creativecommons.org/licenses/by-nc/4.0/).

Volume & Issue: دوره 13 - شماره 1

Title: بررسی میزان هم‌رویدادی و شدت کنش‌پریشی دهانی و کلامی در بزرگسالان مبتلا به ضایعه مغزی نیمکره چپ

http://rehabilitationj.uswr.ac.ir/article-1-927-fa.html

هدف: کنش‌پریشی کلامی و دهانی، نشانگر اختلال برنامه‌ریزی حرکات دهانی کلامی و غیر کلامی هستند. مطالعۀ آنها به فهم چگونگی کنترل حرکتی گفتار کمک می‌کند. هدف تحقیق حاضر شناسایی بیماران دارای کنش‌پریشی دهانی و کلامی، میزان هم رویدادی و شدت آن بود. روش بررسی: در این مطالعۀ غیر تجربی، ۵۵ بزرگسال شامل۲۲ زن و ۳۳ مرد با دامنه سنی ۲۳ تا ۸۴ سال و دارای ضایعۀ مغزی چپ به روش در دسترس انتخاب و با آزمایه‌های کنش‌پریشی کلامی و کنش‌پریشی دهانی آزمایش و فیلم برداری شدند. سه آسیب شناس گفتار و زبان به صورت مستقل شدت کنش‌پریشی را نمره گذاری کردند. تحلیل داده‌ها با آزمون تی مستقل، ضریب همبستگی پیرسون، ضریب همبستگی فی و ضریب توافقی سی و با استفاده از نرم افزارSPSS  نسخۀ ۱۲ انجام شد. یافته‌ها: میانگین کنش‌پریشی دهانی و کلامی در افراد دارای و بدون کنش‌پریشی دهانی و کلامی تفاوت معنادار نشان داد (۰/۰۰۱>P) و ۴۲ بیمار هر دو نوع کنش‌پریشی را با همبستگی معنادار نشان دادند (r=0/75, P<0/001) نفر بدون کنش‌پریشی و ۷ نفر فقط یک نوع کنش‌پریشی داشتند. مقایسۀ هم رویدادی اختلالات (۰/۵۹=Phi) و شدت‌های مختلف کنش‌پریشی دهانی و کلامی (۰٫۶۸=C) نشانگر توافق نسبتاً بالا بود (۰/۰۰۱>P).  نتیجه‌گیری: تحقیق حاضر هم رویدادی بالای اختلالات کنش‌پریشی دهانی و کلامی را آشکار ساخت که نشان می‌دهد کنترل حرکتی گفتار تحت تاثیر کنترل حرکتی عمومی‌تر کلامی و غیر کلامی قرار دارد. .

Title: بررسی تغییرات شیب تابع رشد دامنه پتانسیل عمل مرکّب الکتریکی طیّ سه ماه بعد از دریافت پردازشگر گفتار در کودکان کاشت حلزون‌شده

http://rehabilitationj.uswr.ac.ir/article-1-475-fa.html

هدف: در تله متری پاسخ عصبی، تحریک و ثبت پاسخ عصب شنوایی توسط الکترودهای درون حلزونی پروتز کاشت حلزون صورت می‌پذیرد. در این جا تحریک الکتریکی توسط یک الکترود به عصب شنوایی ارائه و پاسخ حاصله که پتانسیل عمل مرکب الکتریکی نامیده می‌شود، توسط یکی از الکترودهای مجاور ثبت می‌گردد. مهم‌ترین کاربردهای بالینی این آزمون عبارتند از ارزیابی و پایش پاسخ‌های عصب شنوایی در حین و بعد از عمل جراحی و کمک به تنظیم اولیه پردازشگر گفتار. هدف این مطالعه، ارزیابی تغییرات شیب تابع رشد دامنه این پتانسیل طی سه ماه بعد از دریافت پردازشگر گفتار در کودکان کاشت حلزون شده بود. روش بررسی: در این مطالعه طولی با استفاده از آزمون فریدمن، تغییرات شیب تابع رشد دامنه این پتانسیل طی چهار جلسه پس از دریافت پردازشگر گفتار و با فواصل زمانی تقریباً یک ماهه، در الکترودهای اول، هشتم، پانزدهم و بیستم در تمامی کودکانی که در تابستان و پاییز سال 1386 در بیمارستان‌های امیر اعلم و حضرت رسول اکرم(ص) تهران تحت عمل جراحی کاشت حلزون قرار گرفته بودند، بررسی شد. یافته‌ها: میانگین شیب‌ها، در ادامه کار بیش از جلسه اول بوده و مقایسه بین الکترودی نتایج حاکی از کاهش معنی دار میانگین شیب تابع رشد دامنه در الکترودهای رأسی‌تر نسبت به الکترود اول بود (۰/۰۵>P). نتیجه‌گیری: ثبات نتایج حاصله می‌تواند باعث اطمینان خاطر بیشتر درمانگر جهت تنظیم پردازشگر گفتار برای مدتی نسبتاً طولانی گردد. هم چنین نتایج بهتر الکترودهای رأسی‌تر نیز می‌توانند موجب ارائه و اعمال یک راهکار کدگذاری مؤثرتر گردند.

Title: اعتباریابی مقیاس مداخله خانواده-محور برای مادران با کودک دارای نیازهای ویژه

http://rehabilitationj.uswr.ac.ir/article-1-751-fa.html

هدف: هدف این پژوهش اعتباریابی مقیاس مداخله خانواده- محور(FFIS) برای مادران با کودک دارای نیازهای ویژه بود. روش بررسی: پژوهش حاضر یک بررسی توصیفی- پیمایشی از نوع ابزارسازی و اعتبارسنجی بود. بدین منظور 200 نفر از مادران به روش نمونه‌گیری در دسترس از مراکز آموزشی کودکان با نیازهای ویژه انتخاب شدند. پرسشنامه‌ی پژوهش شامل، مقیاس مداخله خانواده- محور(FFIS) بود. به منظور بررسی نتایج پژوهش با استفاده از نسخه 16 نرم افزار اس. پی. اس. اس از ضریب همبستگی، آلفای کرونباخ، آزمون- بازآزمون، همبستگی گویه- نمره کل و تحلیل عامل استفاده گردید. یافته‌ها: بررسی همخوانی درونی مقیاس مداخله خانواده- محور(FFIS) نشان داد که تمام 40 گویه این مقیاس با نمره کل همبستگی بالایی داشتند، آلفای کرونباخ برای کل مقیاس برابر ۰/۸۳ بود. محاسبه ضریب آزمون- بازآزمون به فاصله یک ماه مطلوب و بالا بود. ضریب همبستگی گویه- نمره کل مطلوب و مناسب بود(۰/۰۰۱>P). همبستگی هر یک از زیر مقیاس‌ها با یکدیگر و خرده مقیاس (نمره کل) مطلوب و رضایت بخش بود (۰/۶۴-۰/۱۱=r). نتایج تحلیل عامل با استخراج 5 عامل؛ نظام درگیری، اطلاعات کودک، فعالیت‌های آموزشی خانواده، مساعدت فردی- خانوادگی و مساعدت از امکانات با ارزش بزرگتر از یک در مجموع ۵۵/۸۲٪ از واریانس کل مقیاس را تبیین می کرد. نتیجه‌گیری: نتایج مطالعه حاضر نشان می دهد، نسخه فارسی مقیاس مداخله خانواده- محور(FFIS) می تواند به عنوان یک پرسشنامه مرتبط و قابل قبول همراه با تکرار پذیری بالا جهت سنجش خدمات مداخله مورد استفاده قرار گیرد. یافته‌های این پژوهش نشان دهنده ارزش معنی دار این مقیاس برای استفاده از آن در پژوهش‌های آتی است.

Title: ارتباط نظریه ذهن با اجتماعی‌شدن، مهارت‌های ارتباطی و مهارت‌های زندگی دانش‌آموزان دختر و پسر کم‌توان ذهنی

http://rehabilitationj.uswr.ac.ir/article-1-720-fa.html

هدف: هدف این پژوهش بررسی رابطه نظریه ذهن با اجتماعی شدن، مهارت‌های ارتباطی و مهارت‌های زندگی دانش آموزان کم توان ذهنی است. روش بررسی: در این پژوهش که به روش همبستگی است، 60 دانش آموز کم توان ذهنی (30 دختر و 30 پسر) شرکت داشتند. برای جمع آوری داده‌های مربوط به نظریه ذهن از تست نظریه ذهن 38 سؤالی استفاده شد و داده‌های مربوط اجتماعی شدن، مهارت‌های ارتباطی و مهارت‌های زندگی با استفاده از مقیاس رفتار انطباقی واینلند گردآوری شد. یافته‌ها: رابطه بین نظریه ذهن سطح دوم و اجتماعی شدن به لحاظ آماری معناداری است (۰/۰۰۵=P) ولی رابطه آماری معناداری بین سطوح نظریه ذهن با مهارت‌های ارتباطی و مهارت‌های زندگی مشاهده نشد (۰/۰۵). نتیجه‌گیری: با افزایش درک باور غلط اولیه (نظریه ذهن سطح دوم)، اجتماعی شدن دانش آموزان کم توان ذهنی افزایش می‌یابد.

Title: کودکان دچار معلولیت و انجام تمرینات توان‌بخشی در منزل: موانع پیروی از توصیه‌های درمانگران

http://rehabilitationj.uswr.ac.ir/article-1-830-fa.html

هدف: انجام تمرینات توانبخشی توصیه شده درمانگران در منزل نقش مهمی در موفقیت برنامه توانبخشی کودکان معلول ایفاء می کند. هدف از انجام این مطالعه تعیین موانع پیروی والدین کودکان معلول از انجام تمرینات توانبخشی در منزل بود. روش بررسی: در این مطالعه پیمایشی، والدین همه 60 کودک معلول که در مراکز سرپایی توانبخشی شهر بیرجند در مهرماه 1388 خدمات کاردرمانی، گفتاردرمانی و فیزیوتراپی دریافت می کردند، مورد مصاحبه قرار گرفتند. داده‌ها با استفاده از پرسشنامه نیمه ساختار یافته محقق ساخته و اعتبار یابی شده ای که شامل سوالاتی در مورد «میزان پیروی» و «21 مانع پیروی از انجام تمرینات در منزل» بود، جمع آوری شد، برای تحلیل نسبت شانس عدم پیروی از توصیه‌ها در مواجهه با هر یک از موانع از  آزمون کای دو و تست دقیق فیشر و برای سنجش ارتباط بین تعداد موانع با پیروی از انجام تمرینات از آزمون تی مستقل استفاده شد. یافته‌ها: چهار عامل« نداشتن وسایل انجام تمرینات در منزل» (۰/۰۰۳=P)، «راغب نبودن کودک به انجام تمرینات و عدم تمایل مراقب به ناراحت کردنش» (۰/۰۲۴=P)، «نگرانی مراقب از احتمال آسیب رساندن به کودک ضمن انجام تمرینات» (۰/۰۲۷=P) و «نداشتن مهارت کافی برای انجام تمرینات در منزل» (۰/۰۴۷=P) خطر عدم پیروی والدین از توصیه انجام تمرینات در منزل را به میزان معناداری بالا می برد. بین تعداد موانع درک شده با پیروی از توصیه انجام تمرینات در منزل نیز ارتباط مستقیم معنادار وجود داشت (۰/۰۰۸=P). نتیجه‌گیری: به نظر می‌رسد با طراحی تمرینات به گونه ای که کودک آن را دوست بدارد و تلاش در راه کمک به والدین در کسب ابزار، دانش و مهارت لازم بتوان پیروی والدین از تمرینات منزل را ارتقاء بخشید.

Title: بررسی مقایسه‌ای ارتباط بین عملکرد حرکتی و توجه پایدار در کودکان مبتلا به اختلال نقص توجه-بیش‌فعالی و کودکان عادی 7 تا 10 سال

http://rehabilitationj.uswr.ac.ir/article-1-795-fa.html

هدف: این مطالعه ارتباط عملکرد حرکتی،توجه پایدار و کنترل تکانه را در کودکان مبتلا به اختلال نقص توجه بیش فعالی ((ADHD و عادی بررسی و مقایسه کرده است. روش بررسی: در این پژوهش توصیفی تحلیلی 21 پسر مبتلا به اختلال ADHDو 21 پسر عادی در دامنه سنی 7 تا 10 سال شرکت کردند. عملکرد حرکتی با استفاده از آزمون «کفایت حرکتی برونینکز اوزرتسکی» و توجه پایدار و کنترل تکانه با استفاده از « آزمون عملکرد مداوم» بررسی شد. یافته‌ها: نتایج آزمون تی مستقل و من ویتنی نشان داد گروه ADHD و عادی در عملکردهای حرکتی درشت، ظریف و کلی همچنین توجه پایدار و کنترل تکانه اختلاف معناداری با هم دارند (۰٫۰۰۰۱P) و کلی (۰/۰۰۰۱>P) بین دو گروه تفاوت معناداری دارد. ضریب همبستگی تکانشگری و عملکرد حرکتی درشت(۰/۳۷۹=P)، ظریف (۰/۹۲=P) و کلی (۰/۵۶۲=P) بین دوگروه تفاوت معناداری نداشت. نتیجه‌گیری: بر طبق نتایج مطالعه توجه پایدار و کنترل تکانه با اغلب عملکردهای حرکتی در گروه ADHD و عادی مرتبط بودند این مسئله می‌تواند به درمانگران جهت تعیین اولویتهای توانبخشی و استفاده از راهکارهای دقیق جهت ارتقا عملکرد حرکتی کودکان کمک کند.

Title: مقایسه تأثیر درمان شناختی–رفتاری گروهی و انفرادی بر ترس از ارزیابی مثبت

http://rehabilitationj.uswr.ac.ir/article-1-819-fa.html

هدف: این پژوهش بمنظور بررسی تاثیر درمان شناختی – رفتاری گروهی (CBGT) و انفرادی ( ICBT) در کاهش ترس از ارزیابی مثبت در اختلال اضطراب اجتماعی (SAD) مراجعان به مراکز خدمات روانشناختی انجام شد. روش بررسی: نمونه پژوهش متشکل از 28 نفر بود که بر اساس ملاک مشمول نمره 37 و بالاتر در پرسشنامه فوبیای اجتماعی(SPIN) و بر پایه معیارهای تشخیصی DSM-IV انتخاب و بطور تصادفی 14 نفر (12 نفر اصلی و 2 نفر ذخیره) به گروه CBGT و 14 نفر (12 نفر اصلی و 2 نفر ذخیره) به گروه ICBT تخصیص یافتند. 2 گروه بطور جداگانه تحت 12 جلسه درمانی شناختی-رفتاری گروهی و درمان شناختی رفتاری انفرادی برگرفته از الگوی هایمبرگ قرار گرفتند. یافته‌ها: مقایسه و تحلیل داده های پیش آزمون و پس آزمون پژوهش در پرسشنامه ترس از ارزیابی مثبت (FPES) و پرسشنامه فوبیای اجتماعی (SPIN) و افسردگی بک (نسخه 2 BDI-II) نشان داد که درمان شناختی– رفتاری گروهی پس از کنترل افسردگی از لحاظ تاثیر بر کاهش ترس ار ارزیابی مثبت تفاوت معنی داری با درمان شناختی– رفتاری انفرادی ندارد. بعلاوه، نرخ بهبودی دو گروه CBGT و گروه ICBT به ترتیب برابر 51/41 و 12/32 و فاقد تفاوت معنادار بود. نتیجه‌گیری: معناداری بالینی تفاوت های بدست آمده با ملاکهای مختلف موید برابری تاثیر دو روش مداخله است .

Title: بررسی علل ژنتیکی کم توانی ذهنی اتوزومی مغلوب در استان همدان

http://rehabilitationj.uswr.ac.ir/article-1-866-fa.html

هدف: هدف از این مطالعه بررسی علل ژنتیکی کم‌توانی ذهنی اتوزومی مغلوب در استان همدان است. روش بررسی: در این پژوهش توصیفی-تحلیلی و مقطعی، ابتدا ۷۵ خانواده دارای بیش از یک فرزند کم توان ذهنی با همکاری بهزیستی استان همدان شناسایی و پس از بررسی شجره‌نامه‌ها ۲۵ خانواده با الگوی توارث مفروض اتوزومی مغلوب که در مجموع دارای ۶۰ بیمار (شامل ۳۹ مرد و ۲۱ زن) بودند، انتخاب شدند. کلیه افراد بیمار قبلاً مورد ارزیابی ذهنی و معاینه بالینی قرار گرفته و کم‌توانی ذهنی آن‌ها بوسیله تست سنجش هوش ریون تأیید شده بود. نمونه گیری از تمام افراد مبتلا، پدر، مادر و حداقل دو فرد سالم (در صورت وجود) صورت گرفت. سپس افراد مبتلا به وسیله تست نشانگان ایکس شکننده، آنالیز کروموزومی و تست متابولیک مورد بررسی قرار گرفتند. همچنین در موارد میکروسفالی آنالیز پیوستگی برای هفت جایگاه ژنی شناخته شده کم‌توانی ذهنی اتوزومی مغلوب همراه با میکروسفالی نیز صورت گرفت. یافته‌ها: از بین موارد مورد بررسی ۵ خانواده به نشانگان ایکس شکننده مبتلا بودند. ناهنجاری کروموزومی در هیچ یک از خانواده‌ها مشاهده نشد. یک خانواده دارا نقص متابولیک کمبود آنزیم آسیل کوآ-دهیدروژناز اسیدهای چرب زنجیره متوسط تشخیص داده شد. همچنین در 3 خانواده کم‌توانی ذهنی با میکروسفالی همراه بود که هیچ یک به لوکوس‌های شناخته شده میکروسفالی اولیه (ام.‌سی.‌پی.‌اچ) پیوستگی نداشتند. نتیجه‌گیری: نشانگان ایکس شکننده و کم‌توانی ذهنی اتوزومی مغلوب همراه با میکروسفالی با فراوانی به ترتیب ٪۲۰ و ٪۱۲ از مهم‌ترین عوامل بروز کم‌توانی ذهنی در استان همدان هستند.

Title: مقایسه سبک‌های مقابله‌ای مادران کودکان استثنایی با یکدیگر و مادران کودکان عادی

http://rehabilitationj.uswr.ac.ir/article-1-649-fa.html

هدف: هدف از اجرای این پژوهش مقایسه سبک‌های مقابله‌ای مادران کودکان استثنایی با یکدیگر و همینطور با مادران کودکان عادی بود. روش بررسی: در این پژوهش که از نوع علی و مقایسه‌ای پس رویدادی است، نمونه‌ای به حجم 120 نفر از مادران در 4 گروه کم توان ذهنی، کم بینا، کم شنوا و عادی به روش تصادفی خوشه‌ای در شهرستان کرج انتخاب شدند. پرسشنامه سبک‌های مقابله‌ای لازاروس و فولکمن (1998) در این پژوهش به عنوان ابزار استفاده گردید. داده‌های بدست آمده با استفاده از آمار توصیفی، تحلیل واریانس چند متغیری و آزمون تعقیبی توکی تجزیه و تحلیل شد. یافته‌ها: نتایج نشان داد که از لحاظ استفاده از سبک‌های مقابله‌ای دوری جویی، مسئولیت پذیری، گریز و اجتناب و بازبرآورد مثبت بین مادران کودکان استثنایی با مادران کودکان عادی در سطح 5 درصد تفاوت معناداری وجود دارد. هم چنین این پژوهش نشان داد که بین مادران گروه‌های کودکان استثنایی با یکدیگر هم تفاوت‌هایی وجود دارد که گروه مادران کودکان کم شنوا بیشترین تفاوت رابا دیگر گروه مادران دارا می‌باشند. نتیجه‌گیری: از آنجایی که مادران کودکان استثنایی دراستفاده از سبک‌های مقابله‌ای با یکدیگر و با مادران کودکان عادی تفاوت دارند، بر این اساس پیشنهاد می‌شود، مسئولین و متخصصانی که در این خصوص فعال هستند، راهکارهای مناسب برای مقابله با فشار روانی را به والدین کودکان استثنایی به خصوص مادران به طور جداگانه آموزش دهند. با ارائه آموزش و حمایت‌های بیشتر، مادران می‌توانند با استرس‌های زندگی مناسب تر برخورد کنند و خانواده از آرامش روانی بالاتری بهره مند خواهد شد.

Title: مقایسه دو روش تمرینی بر عملکرد حرکتی و تعادل کودکان مبتلا به فلج مغزی دایپلژی اسپاستیک

http://rehabilitationj.uswr.ac.ir/article-1-836-fa.html

هدف: هدف از این تحقیق بررسی اثر تمرینات بر روی پله و نشستن به ایستادن بر عملکرد حرکتی درشت و تعادل کودکان مبتلا به فلج مغزی دی‌پلژی اسپاستیک و مقایسه این اثرات در خانه و کلینیک است. روش بررسی: در این مطالعه مداخله ای و شبه تجربی، ۲۱ کودک مبتلا به فلج مغزی دی‌پلژی اسپاستیک که به طور مستقل می‌توانستند راه بروند، از جامعه در دسترس انتخاب شدند و به طور تصادفی به سه گروه تقسیم شدند (۷ نفر در گروه تمرین در کلینیک، ۷ نفر در گروه تمرین در منزل و ۷ نفر در گروه کنترل). گروه تمرین درمنزل، فعالیت‌های روزانه زندگی به علاوه یک برنامه تمرینی شامل نشستن به ایستادن و بالارفتن از پله از جهات مختلف را ۵ روز در هفته و به مدت 6 هفته با نظارت والدین انجام داد.گروه تمرین در کلینیک هم همین تمرینات را با نظارت درمانگر در کلینیک توانبخشی انجام داد و گروه کنترل تنها فعالیت‌های منظم روزانه را ادامه داد. در این مطالعه از TUG (زمان بلند شدن و رفتن) و  FRT (تست رساندن عملکردی) به عنوان آزمونهای تعادلی استفاده شد و عملکرد راه رفتن با آزمون ۱۰متر راه رفتن، و عملکرد حرکتی با بخش D و  E آزمون  GMF اندازه گیری شد. یافته‌ها: در پایان دوره تمرین افزایش معناداری در میانگین آزمون رساندن عملکردی FRT در هر دو گروه تمرینی ثبت شد که نسبت به گروه کنترل معنادار بود (۰/۰۵>P)، بعلاوه کاهش معنادار در آزمون  TUGدر گروه تمرین در کلینیک ثبت شد که نسبت به گروه کنترل معنادار بود (۰/۰۵>P). در سایر نتایج اندازه گیری شده، تفاوت معناداری بین سه گروه یافت نشد.  نتیجه‌گیری: تمرین بالا رفتن از پله و نشستن به ایستادن می‌تواند عملکرد تعادلی در کودکان مبتلا به فلج مغزی را بهبود بخشد و تفاوت معناداری بین دو روش تمرینی (تمرین در کلینیک وتمرین در منزل) وجود نداشت.

Title: حقوق کار افراد دارای معلولیت در ایران :از نخستین تلاش‌های بین‌المللی تا کار شایسته

http://rehabilitationj.uswr.ac.ir/article-1-825-fa.html

هدف: تحلیل و مطابقت نظام حقوقی کار افراد دارای معلولیت در ایران با استانداردهای تعیین شدۀ سازمان بین‏المللی کار (ILO) پیرامون کار شایسته. روش بررسی: در این پژوهش توصیفی ـ تحلیلی پس از مطالعۀ تلاش‏های بین‏المللی انجام شده در زمینۀ حقوق کار افراد دارای معلولیت در اسناد عام و خاص حقوق بشری و موازین سازمان بین‏المللی کار، نظام حقوقی کار معلولان در ایران شامل قوانین و مقررات با استفاده از شیوۀ کتابخانه‏ای ـ اسنادی مورد بررسی و نقد قرار گرفته است. یافته‏‌ها: در حوزۀ حقوق بنیادین کار، نظام حقوقی ایران: 1ـ در خصوص کار اجباری معلولین همچون سایـر کارگران دارای ضمانت اجرای کافی بـوده و خلایی از ایـن منظر احساس نمی‏‏نماید. 2ـ در موضوع آزادی روابط سندیکایی نیز کمبودی نداشته و هیچ گونه محدودیتی برای عضویت کارگران یا کارفرمایان معلول در تشکل‏های صنفی موجود مشاهده نمی‏شود. 3ـ در زمینۀ تساوی مزد، هرچند آیین‏نامۀ خاص مربوطه هنوز تصویب نشده اما در مـادۀ 41 قانون کار، حداقل مزد برای کارگـران دارای معلولیت و غیر آن یکسان تعیین شده است. 4ـ در مورد منع تبعیض هم به موجب مبانی قانونی موجود، هرگونه تبعیض به جهت معلولیت ممنوع می‏باشد. در عرصۀ اشتغال نیز اخیراً با تصویب قانون جامع در سال 83 و الحاق به کنوانسیون افراد دارای معلولیت در سال 87، گام‏های جدی در زمینۀ اشتغال معلولین برداشته شده است. خاصّه آنکه فاصلۀ معنادار تسهیلات ارائه شده در حوزه‏های سهمیه‏بندی استخدام بخش دولتی، خدمات آموزشی مستقیم و غیرمستقیم جهت ورود به بازار کار و خوداشتغالی به معلولین خاص انقلاب اسلامی و جنگ تحمیلی (جانبازان گرانقدر) و معلولین عادی ـ مادرزاد یا افرادی که بعد از تولد دچار معلولیت شده‏اند ـ کمتر شده است. نتیجه‏‌گیری: براساس یافته‏های پژوهش تطبیقی حاضر، مبانی قانونی حقوق بنیادین کار معلولین در ایران در همسویی حداکثری با حداقل استانداردهای کار شایسته بوده و از این رو به نظر می‏رسد این مهم با گذر از مرحلۀ تقنین، نیازمند نظارت شایسته براجرای نظامات موجود است. در عرصۀ اشتغال نیز قدم‏های نخست تقنینی اولاًـ در چارچوب سیاستگذاری جامع و رفع هرگونه تبعیض منفی و از قضا برقراری تبعیض مثبت با الحاق به کنوانسیون افراد دارای معلولیت. ثانیاًـ در بستر قانون‏گذاری خاص و مصداقی با تصویب قانون جامع برداشته شده و البته نیازمند تکمیل و پیگیری در اجراست.

Title: تأثیر کوتاه‌مدت دو نوع تمرین ثبات‌دهنده ستون فقرات بر آزمون‌های تعادلی و محدوده ثباتی پویا در مردان مبتلا به کمردرد مزمن غیراختصاصی

http://rehabilitationj.uswr.ac.ir/article-1-698-fa.html

هدف: هدف پژوهش حاضر، بررسی اثرات کوتاه مدت دو نوع تمرین ثباتی «داخل کشیدن شکم» و تمرین «انقباض همزمان عضلات شکم» روی پارامتر‌های سیستم ثباتی بیودکس در مردان مبتلا به کمردرد غیراختصاصی تکرارشونده بود. روش بررسی: این تحقیق از نوع کارآزمایی بالینی و به صورت موردی-شاهدی بر روی ۳۰ مرد مبتلا به کمردرد غیراختصاصی تکرار شونده که به صورت ساده و در دسترس انتخاب و تصادفی به ۳ گروه کنترل (۱۰ نفر) و ۲ گروه مورد (۱۰ نفر) تقسیم شدند، انجام گرفت. قبل و بلافاصله بعد از تمرینات، شاخص‌های ثباتی تعادل دینامیک (کلی، قدامی-خلفی و داخلی-خارجی) و محدوده ثباتی پویا توسط سیستم ثباتی بیودکس اندازه گرفته شد. داده‌های حاصل با استفاده از آنالیز متقاطع و تحلیل واریانس یک طرفه و آزمون دانشجویی مورد تجزیه و تحلیل آماری قرار گرفت. یافته‌ها: نتایج ما نشان داد که غیر از شاخص داخلی- خارجی، افراد گروه مورد نسبت به گروه کنترل، در شاخص‌های تعادلی بهبود نشان دادند. در گروهی با انجام تمرین «انقباض همزمان عضلات شکم»، در هر دو سطح سفتی ۸ و ۳، کاهش شاخص‌ها، معنادار بود (۰/۰۵>P) . محدوده ثباتی پویا، در هر دو گروه مورد با تمرین فرو بردن شکم (۰/۰۰۱=P) وتمرین هم انقباضی شکم (۰/۰۱=P) افزایش معناداری داشت. نتیجه‌گیری: طبق یافته‌های این مطالعه، بازآموزی یک جلسه ای، بر روی بهبود فعالیت عضلات و متعاقب آن، بهبود تعادل تأثیرگذار است.

Title: بررسی اثربخشی اجتماع درمان‌مدار بر عزت نفس و کاهش عود

http://rehabilitationj.uswr.ac.ir/article-1-777-fa.html

هدف: در سالهای اخیر اجتماع درمان مدار به طور وسیع برای درمان اعتیاد به کار رفته است و اثر بخشی آن مورد مطالعه قرار نگرفته است بنابراین هدف این پژوهش این است که تأثیر درمانی آن را بر افزایش عزت نفس و کاهش عود افراد وابسته به مواد نشان دهد. روش بررسی: این پژوهش بر اساس طرح نیمه تجربی پیش‌ آزمون-پس آزمون بود. در این مطالعه ۱۶۵ فرد مبتلا به اختلال سوء مصرف مواد به شیوه نمونه گیری خوشه‌ای تصادفی از مرکز اجتماع درمان مدار تهران، مشهد انتخاب شدند. آزمودنیهای پژوهش در سال 1386 به مدت 3 تا 6 ماه تحت درمان و یکسال پس از درمان پیگیری شدند. ابزار گردآوری داده‌ها پرسشنامه عزت نفس کوپراسمیت و آزمایش عدم اعتیاد بود. تحلیل آماری به وسیله آزمون آماری تی زوجی انجام شد. یافته‌ها: نتایج تحلیل داده‌ها تفاوت معناداری را در گروه مداخله قبل و بعد از مداخله اجتماع درمان مدار را در افزایش عزت نفس نشان داد (۰/۰۰۱>P). همچنین مداخله اجتماع درمان مدار در کاهش عود افراد مبتلا به اختلال سوء مصرف مواد مؤثر بود. نتیجه‌گیری: یافته‌های این مطالعه نشان داد TC می‌تواند در افزایش عزت نفس، بهبود اختلال سوء مصرف مواد و پیشگیری از عود معتادان مؤثر باشد.
